# Stomatal regulation, leaf water relations, and leaf phenology are coordinated in tree species from the Sonoran Desert

**DOI:** 10.1093/aobpla/plaf041

**Published:** 2025-08-19

**Authors:** Ginna Esperanza Fernández-Molano, Rodrigo Méndez-Alonzo, Mariana Alvarez-Añorve, Teresa Terrazas, Clara Tinoco-Ojanguren

**Affiliations:** Instituto de Ecología, Departamento de Ecología de la Biodiversidad, Universidad Nacional Autónoma de México, Campus Hermosillo, Luis Donaldo Colosio s/n, Los Arcos, Hermosillo, Sonora CP 83250, México; Posgrado en Ciencias Biológicas, Universidad Nacional Autónoma de México, Unidad de Posgrado, Edificio D, 1° Piso, Circuito de Posgrados, Ciudad Universitaria, Coyoacán CP 04510, Ciudad de México, México; Departamento de Biología de la Conservación, Centro de Investigación Científica y de Educación Superior de Ensenada, Carretera Ensenada-Tijuana No. 3918, Zona Playitas, Ensenada, Baja California CP 22860, México; Facultad de Estudios Superiores. Iztacala, Universidad Nacional Autónoma de México. Avenida de los Barrios Numero 1. Col. Los Reyes Ixtacala, Tlanepantla de Baz, CP 54090, Estado de México, México; Instituto de Biología, Universidad Nacional Autónoma de México, Circuito Zona Deportiva S/N, Ciudad Universitaria, Coyoacán CP 04510, Ciudad de México, México; Instituto de Ecología, Departamento de Ecología de la Biodiversidad, Universidad Nacional Autónoma de México, Campus Hermosillo, Luis Donaldo Colosio s/n, Los Arcos, Hermosillo, Sonora CP 83250, México

**Keywords:** abiotic stress, phenology, P–V curves, stomatal conductance, water relations

## Abstract

To cope with heat and water stress, evergreen and deciduous species from hot and arid deserts should adjust their stomatal conductance (*g*_s_) and leaf water potential (Ψ_leaf_) regulation in response to changes in soil water availability, high temperatures, and vapour pressure deficits (VPDs). To test whether phenology induces changes in *g*_s_–Ψ_leaf_ coordination, we tested for associations between 14 leaf traits involved in leaf economics, hydraulics, and stomatal regulation, including minimum seasonal water potential (Ψ_min_) and maximum *g*_s_ (*g*_smax_), turgor loss point (Ψ_tlp_), osmotic potential (Ψ_o_), leaf area (LA), and specific leaf area (SLA), across 12 tree species from the Sonoran Desert with contrasting phenology. We found that foliar phenology, leaf hydraulics, and leaf economic traits are coordinated across species and organized along the axis of physiological efficiency and safety in response to temperature and VPD. Evergreens were more drought-tolerant and more restrictive in water use than deciduous species, maintaining lower *g*_s_ during the rainy season and lower Ψ_min_, Ψ_o_, and Ψ_tlp_. In contrast, deciduous species were less drought-tolerant, shedding their leaves during the dry season. During the rainy season, they exhibit higher *g*_s_ than evergreens, enhancing water transpiration. Moreover, deciduous species, as isohydric plants, showed stricter control over *g*_s_ and finer regulation of leaf water potential (Ψ_leaf_). Due to their remarkable physiological diversity, desert trees can endure extreme environmental conditions by employing contrasting hydrological strategies.

## Teaser

Desert trees have adapted to survive extreme heat and drought by regulating the amount of water they lose through their leaves and managing their internal water content. We studied 12 tree species in the Sonoran Desert. Some of these trees are evergreens, which retain their leaves year-round, while others are deciduous, shedding their leaves during dry periods. Our research revealed that these two groups employ different strategies to withstand harsh conditions. Evergreens conserve water and manage drought by using less water overall. In contrast, deciduous trees avoid drought stress by dropping their leaves in dry times but utilize more water when it is available. Despite their differences, both types of trees exhibit distinct patterns in how their leaf traits work together to balance water use and ensure survival. This diversity of strategies sheds light on how desert trees thrive in such a challenging environment.

## Introduction

In extreme environments, such as the Sonoran Desert (SD), where high temperatures and long-lasting droughts impose severe limits on plant water balance, stomatal regulation becomes a crucial adaptive mechanism for species survival. Stomata function as dynamic control valves, regulating gas exchange and transpiration to maintain water levels while facilitating carbon uptake (Lambers and Oliveira 2019, Brodribb et al. 2020). Stomatal responses to environmental factors, such as vapour pressure deficit (VPD), air temperature, soil moisture, and light, are vital for controlling water loss and leaf temperature, especially under changing and extreme desert conditions (Skelton et al. 2015, Ahrens et al. 2020, Driesen et al. 2020). Stomatal behaviour is closely linked to leaf water status, especially with the water potential at the turgor loss point (Ψ_tlp_), across a hydraulic sequence of traits that proceed as stomatal closure through abscisic acid production (Binstock et al. 2024), followed by reductions in leaf hydraulic conductance, to Ψ_tlp_, and finally, to reductions in stem hydraulic conductivity and, therefore, in hydraulic dysfunction (Bartlett et al. 2016). Traits such as osmotic potential (Ψ_o_), modulus of elasticity (*ɛ*), saturated water content (SWC), and capacitance (*C*) affect how leaves retain or lose water under stress and reflect a species’ capacity to sustain turgor and function during drying conditions. These physiological traits, along with stomatal conductance and water potential thresholds, provide insights into how species adapt to environmental extremes.

To understand how plants control water loss and gas exchange under environmental stress, two physiological parameters are often examined: maximum stomatal conductance (*g*_smax_) and minimum seasonal water potential (Ψ_min_). Maximum stomatal conductance indicates the highest seasonal value of *g*_s_ observed in fully developed, nonsenescent leaves under natural conditions, serving as a measure of stomatal efficiency (Körner 1995, Hoshika et al. 2018, Henry et al. 2019). Conversely, Ψ_min_ represents the most negative leaf water potential measured during the growing season and reflects the plant’s capacity to function under drought conditions (Pivovaroff et al. 2018, Yan et al. 2020). This parameter is closely related to Ψ_close_—the threshold below which stomata close to prevent excessive water loss—making it an important trait for assessing hydraulic safety (Bartlett et al. 2016, Henry et al. 2019). Ψ_min_ also offers insights into a plant’s water status and the maximum tension that its xylem can withstand (Bhaskar and Ackerly 2006, Bartlett et al. 2016), while accounting for environmental factors such as soil moisture and atmospheric demand (Bhaskar and Ackerly 2006). Together, *g*_smax_ and Ψ_min_ provide a helpful framework for exploring the coordination between stomatal regulation and hydraulic strategies across different species.

Species differ in how they regulate their stomatal conductance, which depends on their position along the isohydric–anisohydric continuum (Pivovaroff et al. 2018, González-Rebeles et al. 2021). Isohydric species respond to water stress by closing their stomata early to keep Ψ_leaf_ stable, avoiding hydraulic failure. Anisohydric species, on the other hand, keep their stomata open at lower Ψ_leaf_ levels, tolerating more water loss and a higher risk in exchange for continued carbon uptake (McDowell et al. 2008). These different strategies are often linked to leaf habit: deciduous species usually display isohydric behaviour and drought avoidance, while evergreens show anisohydric behaviour and drought tolerance (McDowell et al. 2008, González-Rebeles et al. 2021). These physiological strategies also align with the leaf economic spectrum (LES), where fast-return species (typically deciduous) maximize short-term gains with high specific leaf area (SLA), low tissue density, and short leaf lifespan, while slow-return species (usually evergreen) invest in durable, resource-conserving traits (Wright et al. 2004, Reich 2014). Therefore, the connection between leaf habit, water relations, and stomatal regulation may form a coordinated set of adaptations that help plants survive high temperatures, limited soil water, and highly desiccant air (i.e. high VPD), in arid systems. While several studies have examined hydraulic traits related to drought and stomatal regulation in different environments (Anderegg et al. 2018, Jin et al. 2023, Nadal et al. 2023), more research is needed on the links among leaf phenology, leaf traits, and leaf water relations in the most extreme natural conditions, such as hot deserts. Understanding the links between plants’ water use, stomatal responses to water and temperature stress, and leaf phenology will shed light on plant survival strategies in deserts and may inform ecological restoration strategies.

The SD, with its pronounced climatic seasonality and high interspecific diversity in leaf habits and drought strategies, provides a unique natural laboratory for examining how species balance water loss and carbon gain under extreme conditions.

In this study, we investigated daily and seasonal stomatal regulation in co-occurring evergreen and deciduous tree species in the southern SD. We measured stomatal conductance (*g*_s_) and leaf water potential (Ψ_leaf_) across natural temperature and moisture gradients, and we derived key hydraulic parameters (*g*_smax_, Ψ_min_, Ψ_tlp_, Ψ_o_, *ɛ*, and SLA). We hypothesized that evergreen and deciduous species would exhibit contrasting physiological strategies reflecting their adaptation to extreme heat and drought: evergreens would follow a more conservative, anisohydric strategy with low Ψ_min_ and high tissue resistance traits, while deciduous species would prioritize efficient gas exchange under favourable conditions but rapidly close stomata under stress. By connecting stomatal behaviour to water relations and leaf economics, our study aims to identify different drought response strategies in desert trees through a trait-based comparative approach, highlighting contrasting leaf phenologies and organizing physiological traits along a water safety–efficiency trade-off. Understanding these relationships is crucial for predicting how species will survive in dryland ecosystems as aridity and temperatures increase.

## Materials and methods

### Study site, species sampling, and variables measured

The study was conducted in the SD region, which is known for its extreme weather conditions. Summer temperatures frequently exceed 40°C in this area, while winter temperatures rarely drop below 0°C. Intra-day temperature fluctuations of up to 20°C are common and may occur throughout the year. Annual precipitation is low and exhibits strong seasonal patterns, with ∼80% of the total rainfall occurring during the monsoon season from July to September (Brito-Castillo et al. 2010). Fieldwork was conducted from 2021 to 2022 at two sampling sites with similar temperature and precipitation patterns. These sites share several tree species while hosting unique species, allowing us to increase tree species diversity in our study. The selected sites were ‘Rancho La Pintada’ (28° 35′ 18′′ N, 110° 57′ 51′′ W; elevation 269 m above sea level) and the ‘Centro Ecológico del Estado de Sonora’ (CEES, 29° 01′ 41′′ N, 110° 57′ 09′′ W; elevation 245 m above sea level). Both field sites are located near Hermosillo, Mexico. CEES is within the city perimeter, while ‘Rancho La Pintada’ is situated 54 km to the south. Both sampling sites had low levels of human disturbance and represented typical SD vegetation. From 2016 to 2022, the mean annual precipitation was 340.6 mm at CEES and 321.5 mm at Rancho la Pintada. The maximum yearly temperatures were 45.7°C for CEES and 45.9°C for Rancho la Pintada, with mean annual maximum VPD of 4.84 and 4.82 kPa, respectively (CESAVE - SIAFESON 2024).

**Figure 1.**
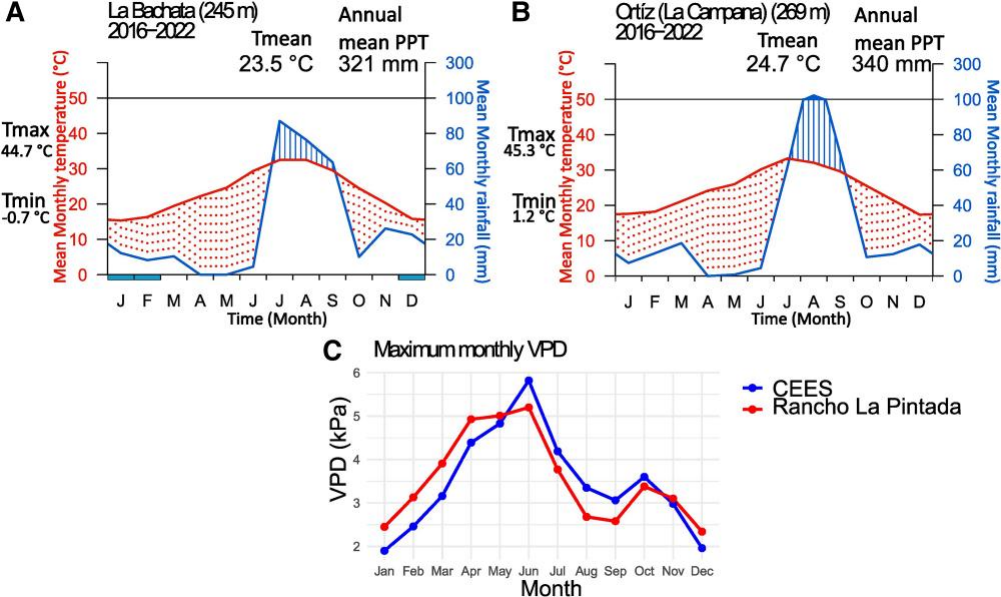
Walter–Lieth climate diagrams (A and B) and maximum VPD (C). The figure shows monthly averages from 2016 to 2022, with data from meteorological stations near each site. Data for “CEES” (A) were obtained from La Bachata meteorological station (REMAS 2024, −111.13190 W 28.96421 N), while data for “Rancho La Pintada” (B) were collected from the Ortiz-La Campana meteorological station (REMAS 2024, − 110.65688 W, 28.30228 N). A–B panels have a dry period (dotted area) when the precipitation plot is positioned below the temperature graph. Otherwise, the period is considered wet (filled in continuous lines).

The SD environmental conditions foster a wide range of plant diversity, as species from the Neotropical and Nearctic biogeographic realms converge in this region (Brito-Castillo et al. 2010). We selected and studied plant species from the SD community characterized by perennial trees and shrubs with small leaves and thorns, often dominated by compound-leaved species (Rzedowski 2006). Perennial species of xeric scrub can be deciduous or evergreen. Deciduous species develop their leaves in summer and lose them at the end of the rainy season, which coincides with summer (July, August, and September; Brito-Castillo et al. 2010) (see Fig. 1). In contrast, evergreen species maintain their leaf canopy throughout the year (Turner et al. 1995, Felger et al. 2001). For our study, we selected 12 tree species (see Table 1), comprising 6 deciduous and 6 evergreen. At each site, we identified and marked five to six trees of each species, ensuring they were mature, with similar heights, and in good health. To assess our hypothesis regarding the influence of different functional and physiological traits on stomatal responses, we measured 14 leaf traits (see Table 2).

**Table 1.** Sonoran Desert tree species included in this study.

N°	Species	Order	Family	Abbreviation	Foliar phenology
1	*Bonellia macrocarpa* subsp. *pungens* (A. Gray) B. Ståhl & Källersjö	Ericales	Primulaceae	Bmp	Evergreen
2	*Bursera fagaroides* (Kunth) Engl.	Sapindales	Burseraceae	Bf	Deciduous
3	*Bursera laxiflora* S. Watson	Sapindales	Burseraceae	Bl	Deciduous
4	*Bursera microphylla* A. Gray	Sapindales	Burseraceae	Bm	Deciduous
5	*Forchhammeria watsonii* Rose	Brassicales	Resedaceae	Fw	Evergreen
6	*Fouquieria macdougalii* Nash	Ericales	Fouquieriaceae	Fm	Deciduous
7	*Guaicum coulteri* A. Gray	Zygophyllales	Zygophyllaceae	Gc	Evergreen
8	*Jatropha cordata* (Ortega) Müll. Arg.	Malpighiales	Euphorbiaceae	Jc	Deciduous
9	*Neltuma glandulosa* (Torr.) Britton & Rose	Fabales	Fabaceae	Ng	Evergreen
10	*Neltuma velutina* (Wooton) Britton & Rose	Fabales	Fabaceae	Nv	Evergreen
11	*Olneya tesota* A. Gray	Fabales	Fabaceae	Ot	Evergreen
12	*Parkinsonia microphylla* Torr.	Fabales	Fabaceae	Pm	Deciduous

Foliar phenology, referenced from Turner et al. (1995) and González-Rebeles et al. (2021). Botanical families follow the APG IV system, and scientific names are sourced from the Tropics (2024) database and WFO (2024).

**Table 2.** Functional traits and parameters related to high VPD and temperature resistance were assessed for trees of the SD.

Symbol	Functional traits	Significance	Unit
*g*_s_	Stomatal conductance	The rate of CO_2_ entering or H_2_O vapour exiting through stomata (Lambers and Oliveira 2019)	mmoles m^−2^ s^−1^
Ψ_leaf_	Leaf water potential	Index of the water status of the plant (Lambers and Oliveira 2019)	MPa
Ψ_min_	Minimum seasonal water potential	The most negative Ψ_leaf_ per species per year (Bhaskar and Ackerly 2006)	MPa
*g*_smax_	Maximum overall stomatal conductance	The highest *g*_s_ measurement in a year for the species (Pivovaroff et al. 2018)	mmoles m^−2^ s^−1^
Ψ_o_	Osmotic potential at full turgor	Represents the concentration of solutes in the cells; it is the Ψ_o_ when the RWC is 100% (Bartlett et al. 2012)	MPa
Ψ_tlp_	Leaf water potential at turgor loss point	The Ψ_tlp_ is the leaf water potential at which the cells lose turgor and become flaccid. The Ψ_leaf_ = Ψ_o_ (Bartlett et al. 2012)	MPa
SWC	Saturated water content	Maximum water content per unit of dry matter (Nadal et al. 2023)	g/g
RWC_tlp_	Relative water content at turgor loss point	Represents the hydration level of the leaf at the Ψ_tlp_ (Bartlett et al. 2012)	%
*ɛ*	The bulk modulus of elasticity	Change in Ψ_p_ over the change in the RWC of the symplast (Bartlett et al. 2012)	MPa
*C*_ft_	Capacitance at full turgor	Change in tissue water volume, given by changes in Ψ_leaf_. This *C* is calculated at full turgor (Sack et al. 2022)	MPa^−1^
*C*_tlp_	Capacitance at turgor loss point	This *C* is calculated at the point of loss of turgor (Koide et al. 1989)	MPa^−1^
LA	Leaf area	Represents the projected leaf blade (Pérez-Harguindeguy et al. 2013)	cm^2^
SLA	Specific leaf area	Indicates the cost of building a cm^2^ of leaf area (Pérez-Harguindeguy et al. 2013)	cm^2^/g
δ^13^C	Leaf carbon isotope	Represents intrinsic water-use efficiency (WUEi, *g*_s_/*A*) (Querejeta et al. 2022)	‰

### Diurnal courses of stomatal conductance and water potential

We conducted daily measurements of *g*_s_ and Ψ_leaf_ between 8:00 and 17:00 h, with 2-h intervals, to determine diurnal variations throughout the year. In each round, we recorded data from four to six species, with three individuals per species. For *g*_s_ measurements, we used healthy, mature leaves that were exposed to sunlight. We measured *g*_s_ with an SC-1 Leaf Porometer (Decagon Devices, Pullman, WA, USA), which was calibrated before each measurement round. In cases where the leaves were small, we used two or three leaves or leaflets to ensure complete coverage over the sensor aperture. Simultaneously, we measure the radiation reaching the leaf using a portable quantum meter (Apogee MQ-100), positioning the sensor at the same angle and direction as the leaf. After the *g*_s_ measurement, we cut the small branch containing the measured leaf and placed it in a plastic bag with moist paper. We then quickly stored them in a cooler with ice, avoiding contact between the ice and the plants with foam, and measured Ψ_leaf_ on-site within 15 min using a Scholander–Hammel type pressure chamber (PMS-1550, Corvallis, USA).

Measurements were taken every 2 weeks during the summer of 2021, focusing on daily courses until deciduous species shed their leaves. Afterward, we continued our measurements for evergreen species every month from October 2021 to May 2022.

### Estimation of maximum stomatal conductance and minimum seasonal water potential

To understand how plants vary in gas exchange rates under varying heat and VPD, two key parameters are considered: maximum stomatal conductance (*g*_smax_) and minimum seasonal water potential (Ψ_min_). *g*_smax_ indicates the highest *g*_s_ in healthy leaves and reflects stomatal efficiency (Körner 1995, Hoshika et al. 2018). Ψ_min_, the most negative leaf water potential during the growing season, reflects a plant’s functionality under drought and is linked to Ψ_close_, the threshold for stomatal closure (Yan et al. 2020, Jin et al. 2023). Ψ_min_ also reveals a plant’s water status and xylem tension limits while considering environmental factors (Bhaskar and Ackerly 2006, Bartlett et al. 2016). Together, *g*_smax_ and Ψ_min_ provide insights into stomatal regulation and hydraulic strategies across species.

### Pressure–volume curve parameters

Pressure–volume (PV) curves were constructed using the ‘bench dry method’ (Koide et al. 1989). We measured at least one small branch from three different individuals per species. Individual leaves were not used because the leaves of the studied species from SD are too small, making it difficult to use the Scholander pressure chamber for measurement. The branches were collected from field sites in the summer of 2022. Upon cutting, they were immediately submerged in distilled water to prevent air from entering the xylem vessels. To minimize transpiration, we covered the samples with dark plastic bags. The samples were rehydrated overnight. The next day, small branches were selected and cut with a fresh razor blade. Each branch averaged ∼6 mm in diameter and 16.1 cm in length. For each branch, we measured the gradual weight loss (in grams) and the decrease of Ψ_leaf_.

To analyse curves and determine traits derived from the PV curve, we utilized the ‘Pressure volume analysis spreadsheet tool’ proposed by Sack et al. (2022). This programme fitted a curve between the inverse of the water potential (−1/Ψ_leaf_) and the relative water content (RWC). We based our measurements on the total RWC* (Sack et al. 2022). The parameters determined from the PV curves were bulk modulus of elasticity (*ɛ*), osmotic potential at full turgor (Ψ_o_), turgor loss point (Ψ_tlp_), and capacitance (*C*).

### Leaf area and specific leaf area

The leaf area (LA) and SLA were determined in one leaf per tree of each species, following the methods described by Pérez-Harguindeguy et al. (2013). LA was calculated using the photographic process: we photographed the lamina with a ruler and then measured the area in cm^2^ using the ImageJ programme. The same leaves were dried in an oven at 70°C for 48 h to determine dry weight. The SLA was calculated using the formula:


$$\mathrm{SLA}=\mathrm{LA}(cm^{2})/\mathrm{Dry}\;\mathrm{weight}\;(g)$$

### Determination of carbon isotopic ratio

We determine the carbon isotopic ratio (δ^13^C) as an indicator of the intrinsic water-use efficiency (WUEi) following the methodology described by Querejeta et al. (2022). Leaves from all species were collected during the summers of 2021 and 2022. The leaves were dried in an oven at 70°C for 48 h and then ground in an agate mortar. Each sample weighing 50 mg was packed in sterilized plastic vials and sent to the Environmental Isotope Laboratory at the Department of Geosciences, University of Arizona, Tucson, Arizona, USA.

### Data analysis

We compared leaf intrinsic traits between deciduous and evergreen species. Before conducting the analysis, we performed a Shapiro–Wilk test to evaluate the normality of the data. We then developed the model and assessed homoscedasticity using the Levene's test and the Breusch–Pagan test (function *bptest* in the package ‘lmtest’, Zeileis and Hothorn 2002). For the traits requiring parametric statistics, we conducted a one-way ANOVA. We used a Welch ANOVA, employing the *oneway.test* with the ‘car’ package (Fox and Weisberg 2019), for traits that do not comply with the homoscedasticity. Additionally, we applied the Mann-Whitney U test (Wilcoxon rank-sum tests) for nonparametric traits using the *wilcox.test* function from the ‘stats’ package. Moreover, the Mann-Whitney U test was used to analyze differences in daily *g*_s_ and Ψ_leaf_ between leaf phenologies. To meet model assumptions, SWC, *ɛ*, *C*_ft_, and LA were transformed using the natural logarithm (ln).

To examine the correlation among the functional traits, we calculated the Pearson correlation coefficient (*r*) and associated *P-*values. The correlation analysis was conducted using the *rcorr* function from the ‘Hmisc’ package (Pivovaroff et al. 2018, Nadal et al. 2023). Before calculating the Pearson correlation, the data were standardized. Next, the Shapiro–Wilk test was used to assess the normal distribution of the data.

To investigate how species were ordinated based on functional traits, we conducted a principal component analysis (PCA). We utilized the *PCA* function from the ‘factoextra’ package (Kassambara and Mundt 2020). Before performing the PCA, all data were standardized, and normality was tested using the Shapiro–Wilk test. All statistical analyses and figures were performed in R, version 4.1.2 (R Core Team 2021).

## Results

### Differences in functional traits based on foliar phenology

**Figure 2.**
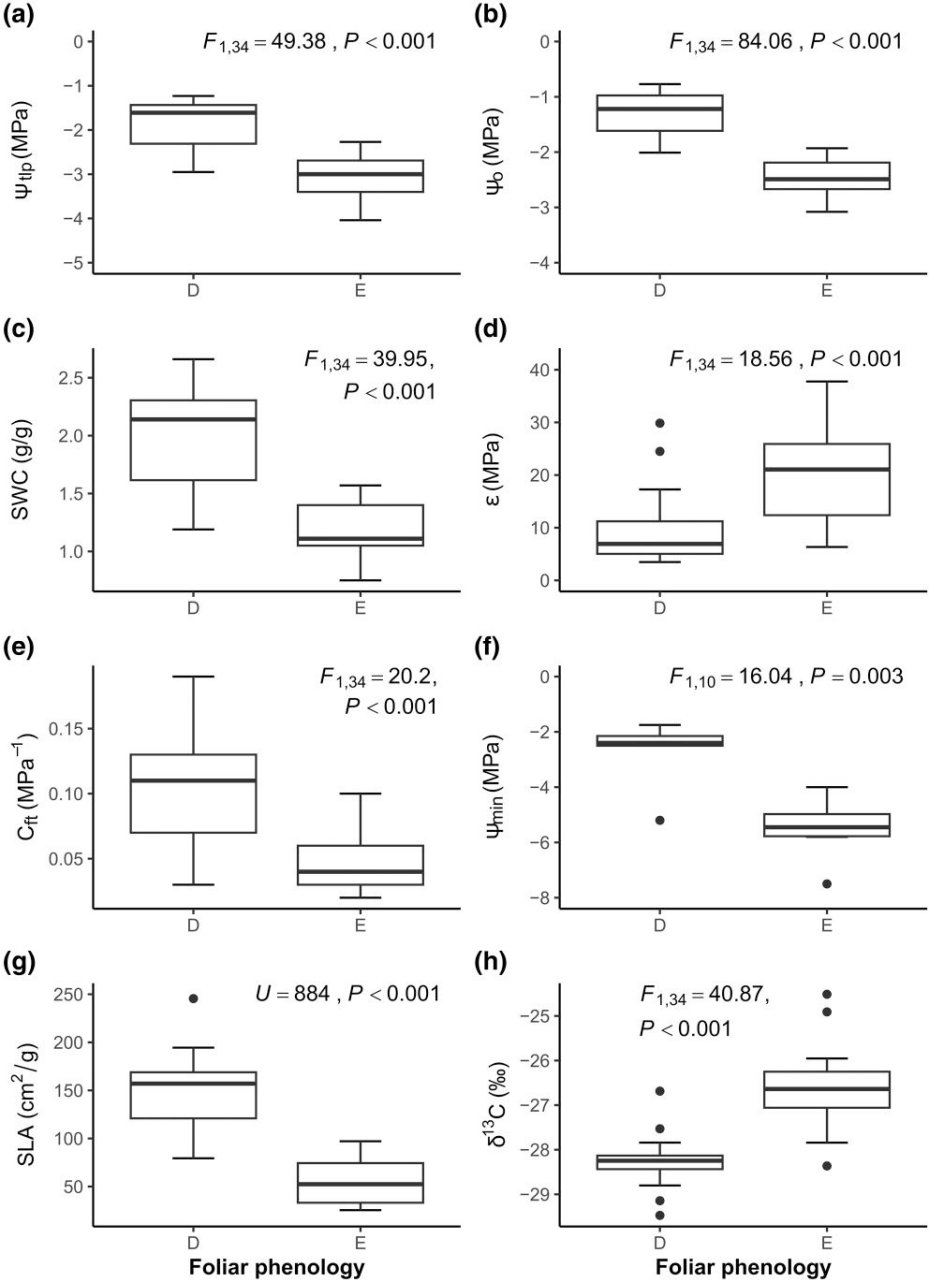
Comparison of intrinsic leaf traits based on leaf phenology for tree species from the SD, Sonora, Mexico. The traits shown are those with significant differences between deciduous (D) and evergreen (E) species. (a) Water potential at the turgor loss point (Ψ_tlp_), (b) osmotic potential at full turgor (Ψ_o_), (c) saturated water content (SWC), (d) bulk modulus of elasticity (*ɛ*), (e) capacitance at full turgor (*C*_ft_), (f) minimum seasonal water potential (Ψ_min_), (g) specific leaf area (SLA), and (h) leaf carbon isotope (δ^13^C). *n* = 6–30 samples per leaf phenology group.

We found differences in leaf traits between evergreen and deciduous species (Fig. 2a–h). Evergreen species exhibited more negative values for Ψ_tlp,_ Ψ_o,_ and Ψ_min_, less negative (higher) δ^13^C, and greater *ɛ* values than deciduous species. Conversely, deciduous species demonstrated higher values of SWC, *C*_ft_, and SLA. Some traits did not show significant differences between the two groups, including *g*_smax_ (*F*_1,10_ = 1.11, *P* > .05), RWC_tlp_ (*F*_1,34_ = 2.47, *P* > .05), *C*_tlp_ (*U* = 221.5, *P* > .05), and LA (*U* = 503.5, *P* > .05).

### Correlations between functional leaf traits

**Figure 3.**
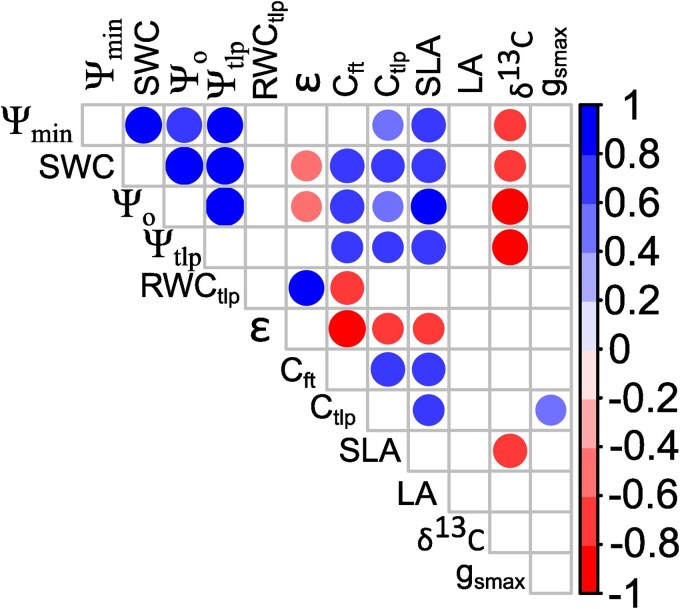
Pearson correlation coefficients among leaf functional traits of 12 tree species from the SD, Sonora, Mexico. Blue circles indicate significant positive correlations (close to 1), while red circles show significant negative correlations (close to −1). Statistical significance was defined as *P* ≤ .05 (*n* = 12). Squares with no circles denote correlations that were not statistically significant.

Minimum water potential showed robust and significant correlations with several traits, including Ψ_tlp_, Ψ_o_, SWC, *C*_tlp_, δ^13^C, and SLA, while *g*_smax_ only correlated with *C*_tlp_ (Fig. 3; Supplementary Fig. S1). Ψ_min_ was positively correlated with Ψ_tlp,_ Ψ_o_, SWC, SLA, and *C*_tlp_ and negatively correlated with δ^13^C. In contrast, *g*_smax_ was positively related to *C*_tlp._ In our analysis, various traits displayed significant correlations. SWC showed positive correlations with Ψ_min_, Ψ_tlp_, Ψ_o_, *C*_tlp_, *C*_ft_, and SLA but a negative correlation with *ɛ*. Similarly, SLA was positively correlated with Ψ_min_, Ψ_tlp_, Ψ_o_, SWC, *C*_tlp_, and *C*_ft_ and negatively correlated with *ɛ*. Ψ_o_, *C*_tlp_, and *C*_ft_ had a negative correlation with *ɛ*, while *ɛ* showed a positive correlation with RWC_tlp_. δ^13^C had a negative correlation with Ψ_min_, Ψ_tlp_, Ψ_o_, SWC, and SLA (Fig. 3).

### Daily patterns of stomatal conductance under natural conditions

**Figure 4.**
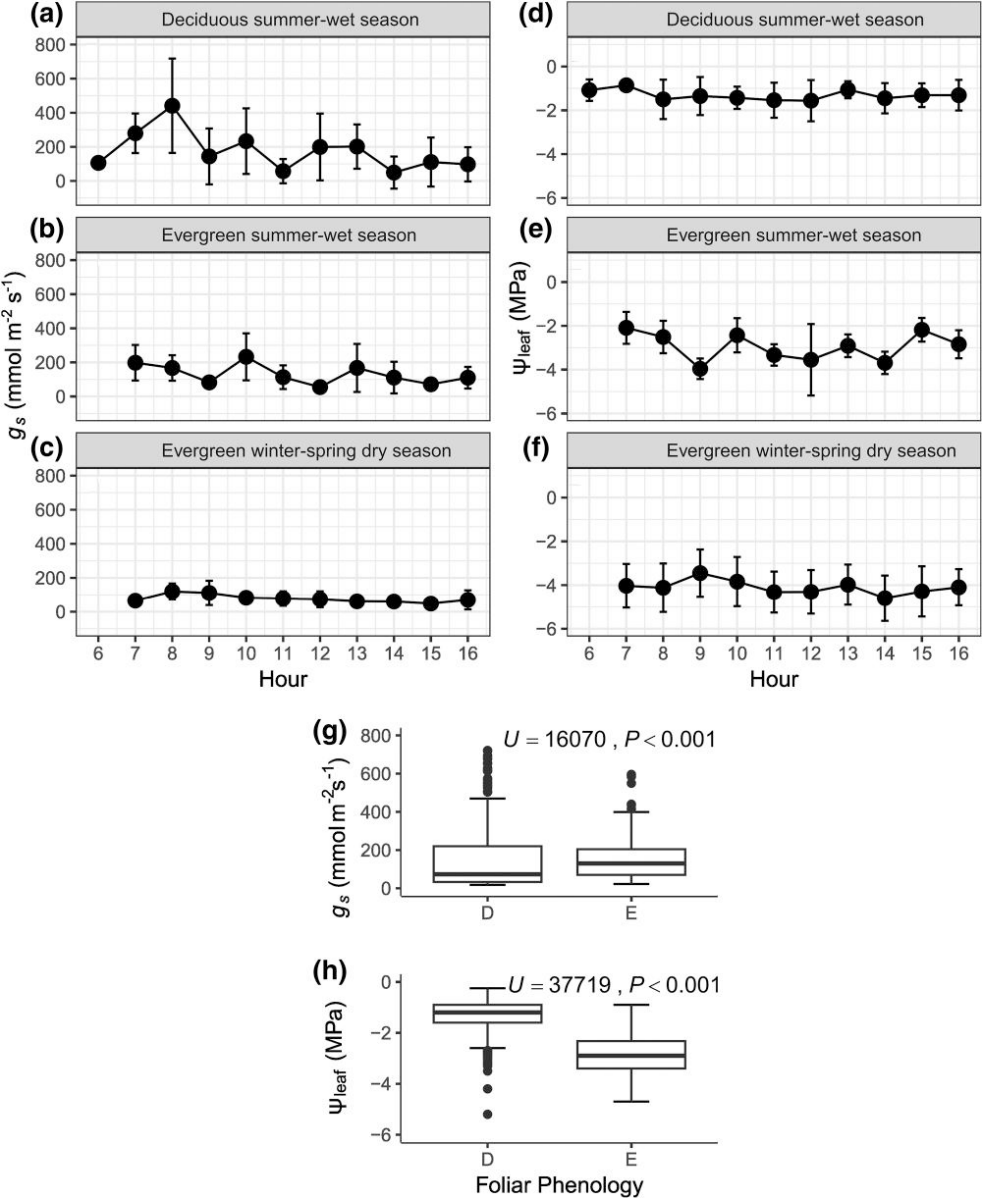
Diurnal patterns of stomatal conductance (*g*_s_) and leaf water potential (Ψ_leaf_) in deciduous (D) and evergreen (E) tree species from the Sonoran Desert. Summer diurnal patterns were measured during the rainy months (August and September). Winter–spring patterns were recorded during the dry months (October–May). (a and b) Diurnal *g*_s_, (c) Diurnal *g*_s_ for evergreen species during the dry season (October–May), (d and e) Diurnal Ψ_leaf_ patterns, and (f) Diurnal Ψ_leaf_ for evergreen species during the dry season. Error bars indicate standard deviation (*n* = 3–39). (g) The distribution of *g*_s_, and (h) the distribution of Ψ_leaf_ for each foliar phenology. The comparison highlights differences in *g*_s_ and Ψ_leaf_ between deciduous (D) and evergreen (E) species.

Diurnal courses of stomatal conductance showed significant differences between phenological groups during the summer when both phenological groups had leaves. The highest *g*_s_ values were observed in the early morning when Ψ_leaf_ was higher and air temperature and VPD were lower. After 9:00, Ψ_leaf_ decreased, while air temperature and VPD increased, leading to a decline in *g*_s_ (Fig. 4a, b, d, and e; Supplementary Fig. S2). Evergreen species maintained lower *g*_s_ and Ψ_leaf_ values during the dry season (Fig. 4c and f). The daily *g*_s_ and Ψ_leaf_ values varied significantly between foliar phenologies (Fig. 4g and h). Evergreens showed lower and less variable *g*_s_ throughout the day, with 92.1% of the *g_s_* measurements ranging from 0 to 300 mmol m^−2^ s^−1^, and only 7.9% exceeding 300 mmol m^−2^ s^−1^. In contrast, deciduous species exhibited more significant variability in *g*_s_, with 82.2% of *g*_s_ values falling within the 0–300 range and 17.8% above 300 mmol m^−2^ s^−1^ (see Supplementary Fig. S3). Significant differences were also observed in the Ψ_leaf_ values of evergreens and deciduous trees (Fig. 4h). For evergreens, 75.5% of Ψ_leaf_ measurements ranged from −4 to −2 MPa, while for deciduous trees, 84.4% were between −2 and −0.25 MPa (see Supplementary Fig. S3).

### Ordination between foliar phenology and functional traits

**Figure 5.**
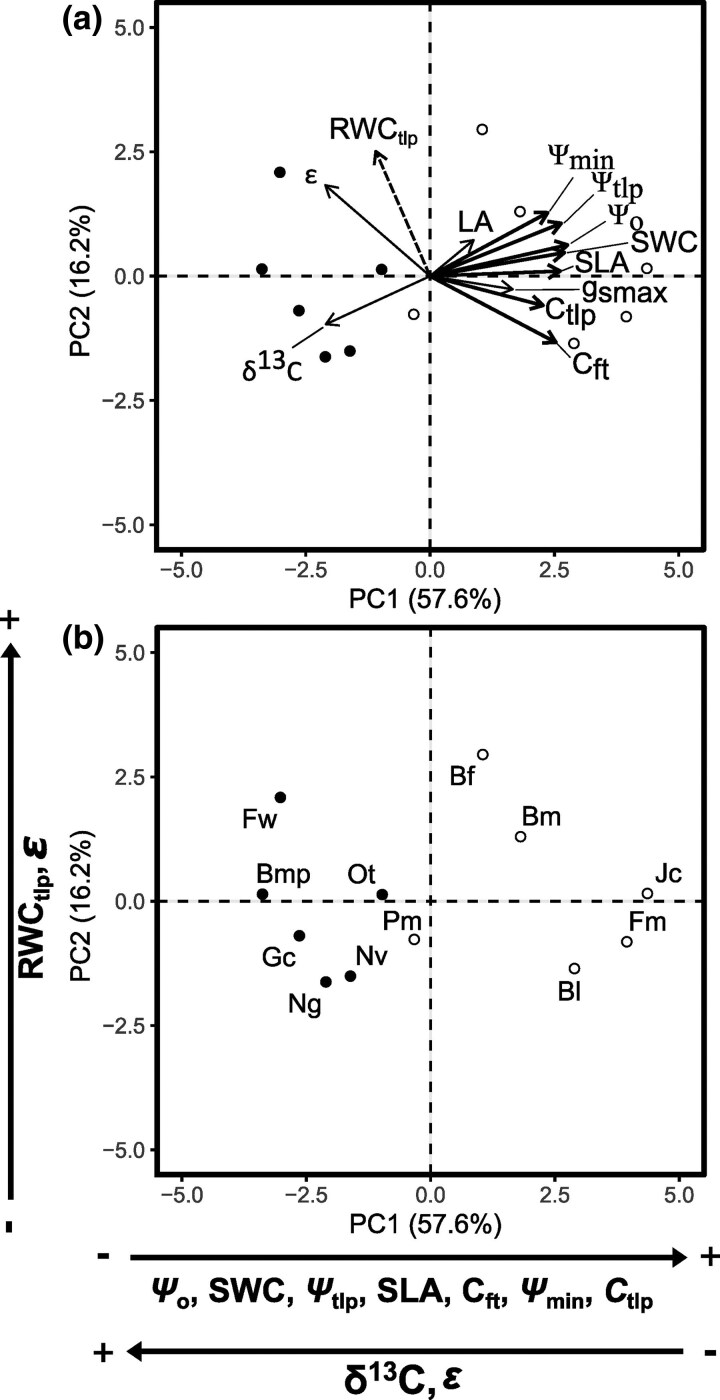
(a) Loading plot and (b) score plot of two axes derived from PCA of 12 leaf functional traits for 12 tree species from the SD. Species acronyms are as in Table 1. Trait acronyms are as in Table 2. In the loading plot (a), thick black lines indicate traits with a loading correlation ≥ 0.8 that contributed to the variance of PC1 (*x*-axis). δ^13^C and *ɛ* were statistically significant and also contributed to the PC1, but with loading correlations of −0.7; therefore they are shown with standard lines. Dashed lines (—) indicate that RWC_tlp_ had a loading correlation ≥ 0.8 and contributed to the variance of PC2 (y-axis). *ɛ* was also statistically significant for PC2, but with a loading correlation of 0.6, and is thus represented with a normal line. Details are summarized in Supplementary Tables S1 and S2. In the score plot (b), black points represent evergreen species, and white points represent deciduous species.

Components 1 (PC1) and 2 (PC2) account for 73.8% of the variability in traits observed in the PCA analysis. The key traits significantly influencing PC1, with a loading correlation ≥0.8, include SWC, Ψ_o_, SLA, *C*_ft_, Ψ_tlp_, *C*_tlp_, and Ψ_min_. Still, δ^13^C and *ɛ* did not have high loadings, but they were statistically significant (Fig. 5a; Supplementary Tables S1 and S2). Species along this axis were further differentiated based on foliar phenology, stomatal regulation, and LES. Deciduous species were positioned on the right side, except for *Parkinsonia microphylla*, which clustered together with the evergreen species on the left side (Fig. 5b). The water cell content traits (SWC, *C*_ft_, Ψ_o_, Ψ_tlp_, and *C*_tlp_) and Ψ_min_ were higher in deciduous compared with evergreens, with SLA also playing a key role in separating these two groups, and δ^13^C and *ɛ* were higher in evergreens than in deciduous species, indicating higher WUEi in evergreens. For PC2, the most significant trait affecting the analysis was RWC_tlp_; however, *ɛ* did not have a high loading, but it was statistically significant (see Supplementary Tables S1 and S2). This axis allowed for the distinction among guilds of species, with those at the bottom exhibiting lower RWC_tlp_ and *ɛ*, contrasting with species found at the top (Fig. 5a and b). This ordination via PCA (Fig. 5) did not account for the assumption of evolutionary nonindependence. Thus, to determine whether evolutionary lineage affects stomatal regulation, we performed a phylogenetic analysis utilizing Pagel and Bloomberg’s phylogenetic signal (see Supplementary Table S3). However, we found no evidence of a phylogenetic signal.

## Discussion

In the SD, where extreme VPDs and high temperatures co-occur with seasonal water availability, stomatal regulation is critical to plant survival. Our study reveals how species with contrasting water-use strategies—deciduous and evergreen—coordinate leaf traits to manage these environmental stressors. Rather than classifying species solely along the iso-anisohydric continuum, we highlight how this functional diversity supports distinct mechanisms of coping with high VPD and temperature in field conditions.

We found functional coordination between stomatal regulation and leaf phenology, as predicted by our hypothesis. Evergreen species exhibited more stable stomatal conductance (*g*_s_), maintaining low *g*_s_ values throughout the year, than deciduous species. In contrast, deciduous species exhibited high variability in *g*_s_, reaching high *g*_s_ values only when the water potential was less negative than −2 MPa, which typically occurred early in the mornings of the rainy season. Most of the time, evergreens showed lower *g*_s_ than deciduous species. These differences in hydraulic behaviour may reflect the distinct stomatal control strategies of these species. Deciduous trees generally have a higher average *g*_s_ during the day but are more stringent in controlling their stomata. They decrease *g*_s_ in response to low Ψ_leaf_, high air temperature, and high VPD, maintaining a higher Ψ_leaf_ and exhibiting isohydric stomatal control (Tardieu and Simonneau 1998). These species take advantage of high water and light availability during the summer season in the SD, increasing *g*_s_. In contrast, evergreens consistently maintain low *g*_s_, even when Ψ_leaf_ is high during summer. They retain low *g*_s_ throughout the rest of the year, displaying traits of anisohydric species. This strategy allows them to tolerate low water potential while maintaining consistent *g*_s_. As a result, they act as a slow resource-use species, even during the rainy summer. Similar patterns have been noted in other dry ecosystems (Brodribb et al. 2003, Méndez-Alonzo et al. 2012, Pivovaroff et al. 2018). Species with lower *g*_s_ had less sensitivity to rising VPD, as our evergreen species; in contrast, species with higher *g*_s_ rates, such as deciduous species, had higher sensitivity to VPD (Oren et al. 1999 ).

Under conditions of high VPD and elevated temperatures, evergreen and deciduous species show distinct physiological strategies related to water potential and leaf traits. Evergreen species demonstrated stronger drought tolerance by consistently having lower values of Ψ_min_, Ψ_o_, and Ψ_tlp_, along with higher leaf elastic modulus (*ɛ*), lower SLA, higher δ^13^C, and tougher leaf tissues. These traits help evergreens maintain leaf function under high VPD and temperature by reducing water loss and preventing turgor loss (Bartlett et al. 2012 , Scoffoni et al. 2014). High *ɛ* values decrease leaf shrinkage and water loss, thereby preserving more water in tissues at Ψ_tlp_ (Bartlett et al. 2012, Scoffoni et al. 2014 ), and allow for partial stomatal opening even during severe drought (Leuschner et al. 2019). The coordination between Ψ_tlp_ and Ψ_min_, also observed in meta-analysis (Jin et al. 2023), suggests an adaptive response to maintain Ψ_leaf_ at a lower level as temperatures rise (Aparecido et al. 2020). Higher δ^13^C values in evergreen species generally indicate higher WUEi (Werner and Máguas 2010), which were observed in our study. The coordination of these traits helps improve stomatal safety and resistance to atmospheric drought and heat in evergreen species (Bartlett et al. 2016, Henry et al. 2019, Marchin et al. 2022, Binstock et al. 2024).

In contrast, deciduous species responded to high VPD and temperature by adopting traits that support water storage and hydraulic buffering. They exhibited less negative Ψ_min_, Ψ_o,_ and Ψ_tlp_ values, consistent with their higher SWC, which allows them to retain more water per unit dry mass (Tyree and Hammel 1972, Nadal et al. 2023). Their greater leaf and stem capacitance (González-Rebeles et al. 2023) enables temporary water storage and release to buffer short-term fluctuations in water availability under high VPD and temperatures (Bartlett et al. 2012, Bucci et al. 2019, Huo et al. 2022). Despite having lower *ɛ* and higher SLA—indicating thinner leaves—deciduous species maintain higher water potentials and maximize gas exchange during favourable periods, achieving greater *g*_s_ and stomatal efficiency in summer (Henry et al. 2019). This strategy reflects an efficient, drought-avoidant approach under thermal and atmospheric stress, supported by the natural coordination of functional traits such as SLA, capacitance, and SWC.

SLA is a key trait used to classify species based on the trade-off between fast and slow resource acquisition within the LES (Wright et al. 2004). In our study, evergreens had lower SLA compared with deciduous species. According to the LES, evergreens invest resources in producing thick, costly leaves with longer lifespans, which results in slower resource acquisition. In contrast, deciduous plants allocate fewer resources to their leaf tissues, leading to thinner, less expensive leaves with shorter lifespans and larger surface areas, which promote faster resource acquisition. Our study shows that SLA predicts various traits related to water-use strategies. We observed a positive correlation between SLA and Ψ_min_, Ψ_tlp_, SWC, Ψ_o_, *C*_tlp_, and *C*_ft_, while a negative correlation was found with *ɛ* and δ^13^C. This reflects the different water-use strategies of the species. Deciduous trees have rapid water resource acquisition and storage capacities that help them avoid water stress. Conversely, evergreens exhibit slower water acquisition and tolerate water stress; however, their traits limit water use when it is available. In this way, differences in LES are linked to VPD and temperature sensitivity, as species focused on fast resource acquisition showed greater sensitivity to increasing VPD than those with slow resource acquisition (Bauman et al. 2022). Furthermore, species with lower LA and SLA demonstrated greater resistance to rising temperatures (Leigh et al. 2017, Sastry and Barua 2017). In conclusion, evergreen species have leaves with higher resistance to increasing VPD and temperatures, allowing them to retain their foliage year-round.

Interestingly, *P. microphylla* is an exception to our hypothesis, as it is deciduous and anisohydric (González-Rebeles et al. 2021). This species exhibited smaller and thicker leaves than other deciduous species, but all cell water relations traits, including Ψ_min_ and *g*_smax_, fell within the range of the evergreen species. Recent research has shown that species from the genus *Parkinsonia* possess photosynthetic stems, with stem conductance and photosynthesis comparable to those of the leaves (Ávila-Lovera et al. 2020). In other words, the stem replaces the function of the leaf without losing significant quantities of water during the periods when the plant is leafless. Thus, *P. microphylla* can be considered somewhat ‘evergreen’ as it maintains active photosynthesis throughout the year but sheds its leaves before the dry season. This suggests that *P. microphylla* may belong to a distinct functional group characterized by species with photosynthetic stems. On the other hand, *Neltuma velutina* is an evergreen phreatophyte. It exhibits evergreen leaf traits, yet it has notably high *g*_smax_ values. This trait may be attributed to its deep root system, which allows it to access groundwater. Higher transpiration rates have been observed in phreatophyte species within the seasonal Amazon forest (Brum et al. 2019).

Our study found no phylogenetic signals in the leaf traits we examined. Previous studies have indicated a lack of phylogenetic signals in traits derived from the P–V curves (Monsiváis-Molina 2018, Ávila-Lovera et al. 2023) as well as in stomatal and foliar traits (Hu et al. 2023), except for stomatal density and stomatal length (Ávila-Lovera et al. 2023). Therefore, our findings suggest that physiological traits display a distinct phylogenetic signal and may be associated with evolutionary convergence processes.

## Conclusion

Our studied species exhibit functional and physiological variability in leaf traits that allow them to cope with extreme environmental conditions in the SD. This interspecific variation supports a spectrum of water-use strategies, reflecting trade-offs between carbon gain, hydraulic safety, and phenological timing.

By integrating field-based measurements of stomatal conductance, water potential, and PV traits, we show that species are organized along an optimal spectrum of equivalently efficient strategies for managing water loss. This spectrum ranges from drought-tolerant, conservative, anisohydric evergreen species to drought-avoiding, fast-acquisition, isohydric deciduous species. These contrasting strategies illustrate that not all drought responses are equivalent: plants use distinct mechanisms—avoidance versus tolerance—to navigate extreme VPD and temperatures, rather than simply falling along an iso-anisohydric continuum. This insight deepens our understanding of plant resilience in the face of intensifying climate extremes.

Further research comparing tree hydraulic and photosynthetic responses, especially under field VPD and temperature stress, will provide mechanistic insights into how desert tree species adjust stomatal behaviour and survive in a rapidly warming world.

## Supplementary Material

plaf041_Supplementary_Data

## Data Availability

The data underlying this article are available in Zenodo: https://doi.org/10.5281/zenodo.16749421.
