# Supplementary material for: Stomatal regulation, leaf water relations, and leaf phenology are coordinated in tree species from the Sonoran Desert
Source: AoB Plants. 2025 Aug 19;17(5):plaf041. doi: 10.1093/aobpla/plaf041 (PMC12406213; doi:10.1093/aobpla/plaf041)
Supplement: plaf041_Supplementary_Data [file plaf041_supplementary_data.pdf]

Supplementary Information for Stomatal regulation, leaf water relations, and leaf phenology are coordinated in tree species from the Sonoran Desert

Authors: Ginna Esperanza Fernández-Molano, Rodrigo Méndez-Alonzo, Mariana Alvarez-Añorve, Teresa Terrazas, and Clara Tinoco-Ojanguren

Methods S1

Phylogenetic analysis

To explore interspecific phylogenetic relationships, a list of the scientific names was made using the IPNI database (International Plant Names Index) and WFO 2023. We analyzed the genus level for species not identified at the species level, treating subspecies as separate species.

We pruned the mega-tree (PhytoPhylo) established for vascular plants (Zanne *et al*. 2014) and subsequently updated by Qian and Jin (2016). This mega-tree is based on the APG III taxonomic classification system.

The tree was generated using the 'S.PhyloMaker' function from the V.PhyloMaker package (Jin and Qian 2019) within the R programming language (R Core Team 2023) to produce the tree.

Phylogenetic signal analysis

Based on the generated phylogenetic tree, we calculated the statistical parameters Pagel's λ and Bloomberg's K. Both statistics operate under the assumption that evolution follows a Brownian model. A high phylogenetic signal suggests that the traits' evolution is influenced by phylogeny, while a low signal indicates that the trait has evolved

independently of phylogenetic relationships. Pagel's λ ranges from 0 to 1, where values close to 1 indicate a strong evolutionary influence on the character, and values near 0 suggest a weak influence. Similarly, Bloomberg's K operates within $1 > K > 0$; values approaching 0 imply that trait evolution is not tied to phylogeny, whereas values close to 1 or exceeding 1 reflect significant phylogenetic inheritance.

We enforced the tree to be ultrametric, ensuring that all branches from the common ancestor have equal lengths, using the "force.ultrametric" function from the "Phangorn" package (Schliep 2011). The trait values were averaged for each species and standardized before analysis.

The phylogenetic signals (λ and K) were calculated using the "Phylosig" function from the "Phytools" package (Revell 2012). This function performs a maximum likelihood test, generating a P-value associated with the phylogenetic signal to test the null hypothesis of λ and $K = 0$ (indicating absence of phylogenetic signal). Our analysis did not reveal any phylogenetic signals for the traits examined.

References

Jin Y, Qian H. 2019. V.PhyloMaker: an R package that can generate very large phylogenies for vascular plants. *Ecography (Cop)* 42:1353–1359. https://doi.org/10.1111/ecog.04434

Qian H, Jin Y. 2016. An updated megaphylogeny of plants, a tool for generating plant phylogenies and an analysis of phylogenetic community structure. *Journal of Plant Ecology* 9:233–239. https://doi.org/10.1093/jpe/rtv047

R Core Team. 2023. *R: A Language and Environment for Statistical Computing*. R Foundation for Statistical Computing, Vienna, Austria. https://www.r-project.org/

Revell LJ. 2012. phytools: An R package for phylogenetic comparative biology (and other things). *Methods in Ecology and Evolution* 3:217–223. https://doi.org/10.1111/j.2041-210X.2011.00169.x

Schliep KP. 2011. phangorn: phylogenetic analysis in R. *Bioinformatics* 27:592–593. https://doi.org/10.1093/bioinformatics/btq706

Zanne AE, Tank DC, Cornwell WK, Eastman JM, Smith SA, Fitzjohn RG, McGlinn DJ, O'Meara BC, Moles AT, Reich PB et al. 2014. Three keys to the radiation of angiosperms into freezing environments. *Nature* 506:89–92. https://doi.org/10.1038/nature12872

Tables

Table S1. Significance of the five principal axes of the PCA analysis of 12 tree species of the Sonoran Desert.

PC	Eigenvalue	%Variance	Cumulative Variance %
**1**	6.91	57.59	57.59
**2**	1.94	16.17	73.75
**3**	1.08	9.03	82.78
**4**	0.98	8.16	90.94
**5**	0.45	3.78	94.72

Table S2. Loadings of PCA analysis of 12 tree species from the Sonoran Desert. The values in bold are the significant values considered on each axis. We considered traits with a weight ≥ 0.8 ecologically relevant. ns = statistically not significant.

N°	TRAIT	PC1	*P*	PC2	*P*
**1**	Ψ_o	**0.94**	< 0.001	0.21	ns
**2**	SWC	**0.92**	< 0.001	0.16	ns
**3**	Ψ_{tlp}	**0.90**	< 0.001	0.36	ns
**4**	SLA	**0.89**	< 0.001	0.03	ns
**5**	C_{ft}	**0.86**	< 0.001	-0.46	ns
**6**	Ψ_{min}	**0.81**	< 0.01	0.43	ns
**7**	C_{tlp}	**0.77**	< 0.01	-0.20	ns
**8**	g_{smax}	0.57	ns	-0.09	ns
**9**	LA	0.30	ns	0.25	ns
**10**	RWC_{tlp}	-0.37	ns	**0.86**	< 0.001
**11**	$\delta^{13}C$	**-0.71**	< 0.01	-0.33	ns
**12**	ε	**-0.72**	< 0.01	**0.62**	0.03

Table S3. Phylogenetic signal for functional traits from trees from the Sonoran Desert, $P \leq 0.05$. Values of (λ) from Pagel's analysis and (K) from Blomberg's analysis.

**Traits**	***λ***	***P***	***K***	***P***
g_{smax}	0.26	0.62	0.47	0.38
Ψ_{min}	0.04	0.97	0.60	0.18
Ψ_{o}	6.61E-05	1	0.41	0.57
Ψ_{tlp}	6.61E-05	1	0.42	0.6
RWC_{tlp}	6.61E-05	1	0.43	0.49
ε	6.61E-05	1	0.45	0.42
C_{ft}	6.61E-05	1	0.34	0.88
C_{tlp}	6.61E-05	1	0.36	0.81
SLA	6.61E-05	1	0.34	0.83
LA	6.61E-05	1	0.46	0.43
$\delta^{13}C$	5.22E-05	1	0.53	0.29

Figures

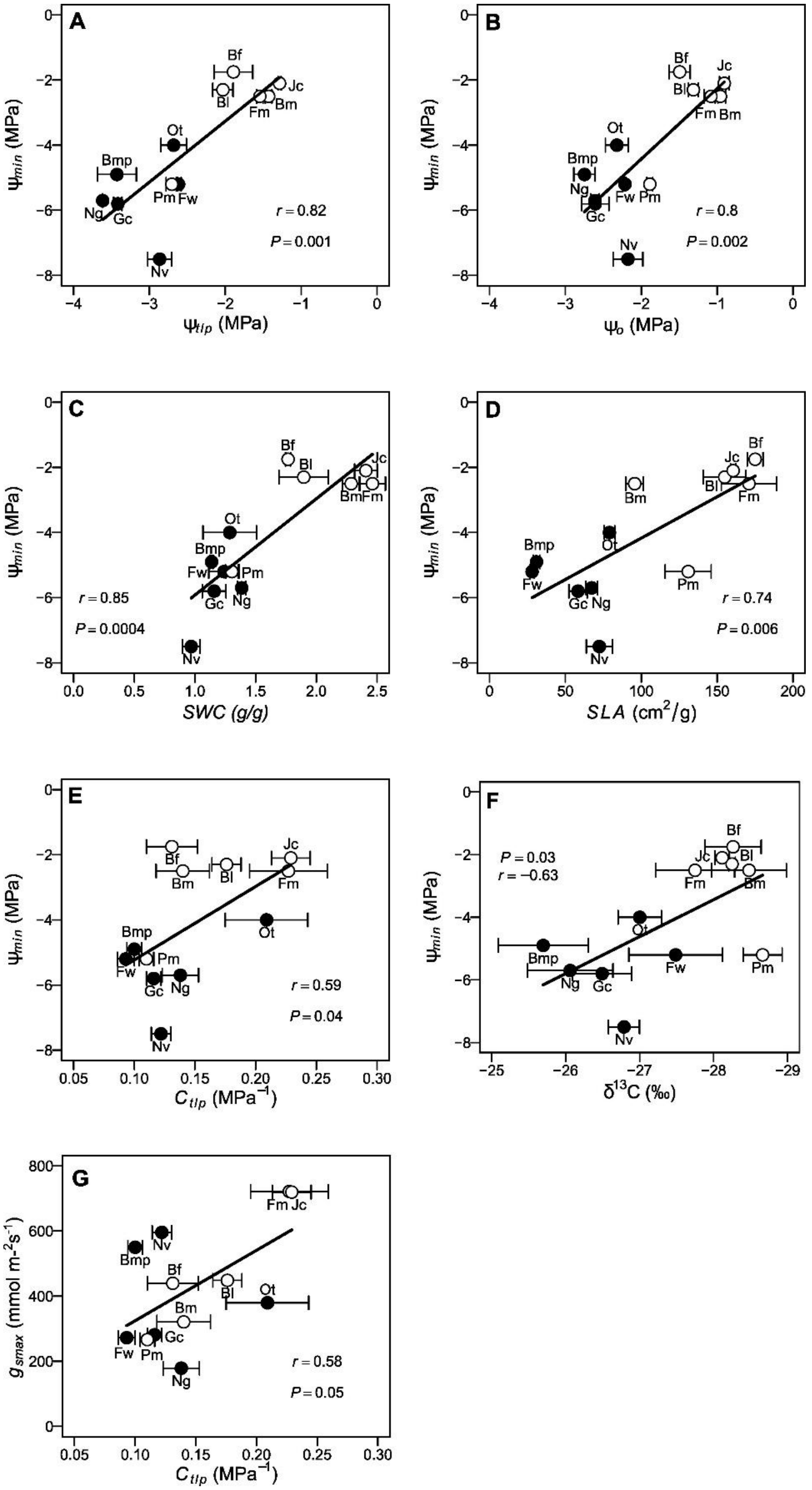
A
r = 0.82
P = 0.001
Ψmin (MPa)
Ψtlp (MPa)
B
r = 0.8
P = 0.002
Ψmin (MPa)
Ψo (MPa)
C
r = 0.85
P = 0.0004
Ψmin (MPa)
SWC (g/g)
D
r = 0.74
P = 0.006
Ψmin (MPa)
SLA (cm2/g)
E
r = 0.59
P = 0.04
Ψmin (MPa)
Ctlp (MPa−1)
F
P = 0.03
r = −0.63
Ψmin (MPa)
δ13C (‰)
G
r = 0.58
P = 0.05
gsmax (mmol m-2s-1)
Ctlp (MPa−1)
Bf
Bl
Bm
Bmp
Fm
Fw
Gc
Jc
Ng
Nv
Ot
Pm

Figure S1. Pearson correlation values of minimum seasonal water potential (Ψ_{min}) and maximum stomatal conductance (g_{smax}) with leaf functional traits in 12 tree species from the Sonoran Desert, Sonora, Mexico. A) Ψ_{min} vs. water potential at turgor loss point (Ψ_{tlp}), B) Ψ_{min} vs. osmotic potential at full turgor (Ψ_o), C) Ψ_{min} vs. Saturated water content (SWC), D) Ψ_{min} vs specific leaf area (SLA), E) Ψ_{min} vs Capacitance at turgor loss point (C_{tlp}), F) Ψ_{min} vs leaf carbon isotope ($\delta^{13}C$), and G) g_{smax} vs C_{tlp}. All points represent the average ± SE (n = 3), except for SLA (n = 5). Ψ_{min} corresponds to the minimum observed value of Ψ_{leaf} per species, and g_{smax} to the maximum g_s per species. Acronyms of species, as in Table 1.

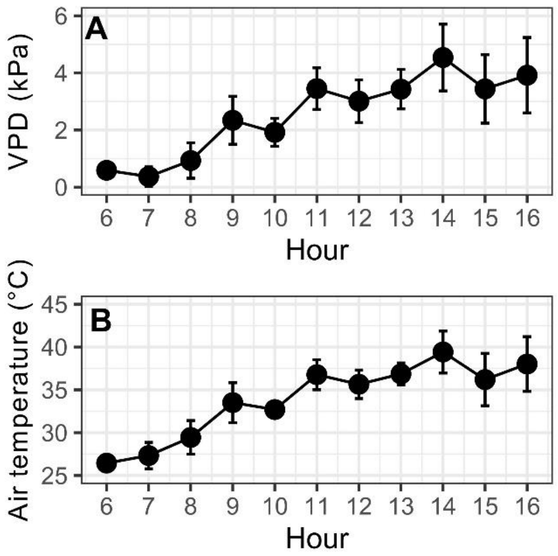

Figure S2. Diurnal variation of vapor pressure deficit (VPD) (A) and air temperature (B) in August and September 2021 in the Sonoran Desert. Average between CEES and "Rancho La Pintada". Error bars represent standard deviation (n = 4–43).

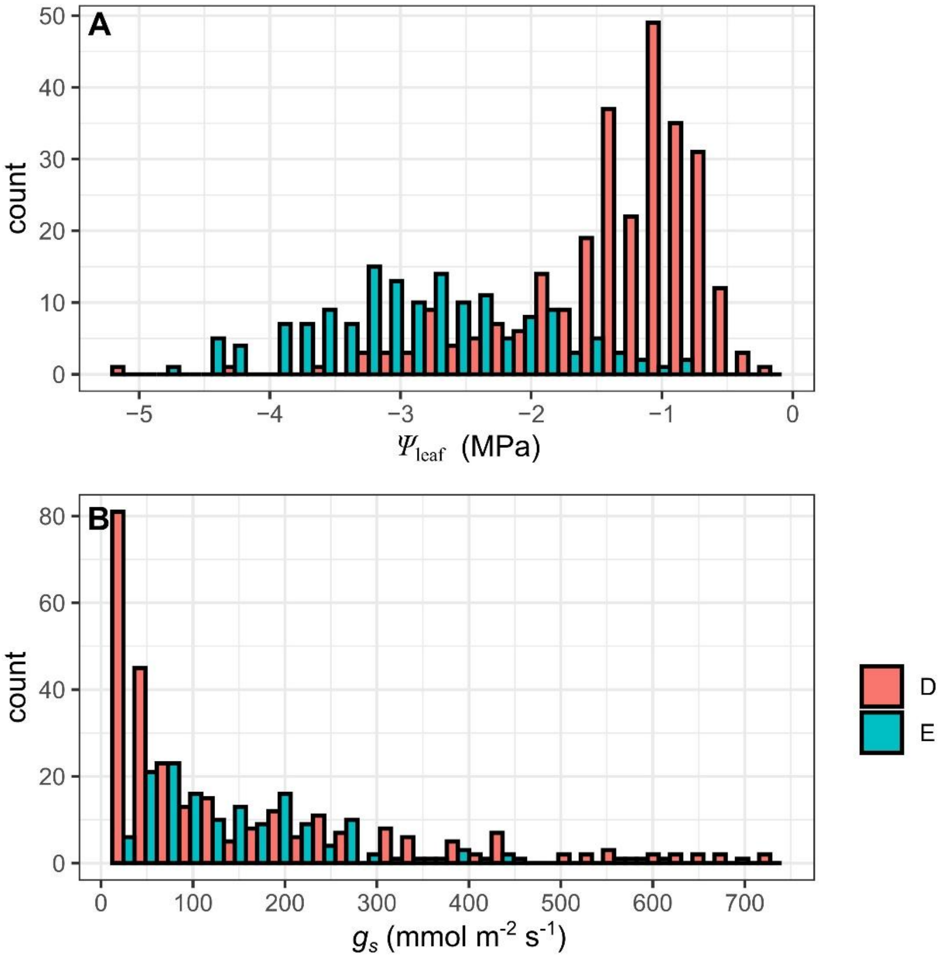

Figure S3. Histograms of A) leaf water potential (Ψ_{leaf}) and B) leaf stomatal conductance (g_s). Data from the diurnal courses of 12 tree species in the summer season from the Sonoran Desert. D = Deciduous, E = Evergreen.
